# Melatonin Therapy Modulates Cerebral Metabolism and Enhances Remyelination by Increasing PDK4 in a Mouse Model of Multiple Sclerosis

**DOI:** 10.3389/fphar.2019.00147

**Published:** 2019-02-28

**Authors:** Majid Ghareghani, Linda Scavo, Yahya Jand, Naser Farhadi, Hossein Sadeghi, Amir Ghanbari, Stefania Mondello, Damien Arnoult, Sajjad Gharaghani, Kazem Zibara

**Affiliations:** ^1^CERVO Brain Research Center, Quebec City, QC, Canada; ^2^Cellular and Molecular Research Center, Yasuj University of Medical Sciences, Yasuj, Iran; ^3^Platform of Research and Analysis in Sciences and Environment (PRASE), Lebanese University, Beirut, Lebanon; ^4^INSERM U 1197, Laboratory of Stem Cells, Transplantation and Immunoregulation, Villejuif, France; ^5^Department of Pharmacology, School of Medicine, Tehran University of Medical Sciences, Tehran, Iran; ^6^Medicinal Plants Research Center, Yasuj University of Medical Sciences, Yasuj, Iran; ^7^Department of Biomedical and Dental Sciences and Morphofunctional Imaging, University of Messina, Messina, Italy; ^8^Oasi Research Institute – IRCCS, Troina, Italy; ^9^Laboratory of Bioinformatics and Drug Design, Institute of Biochemistry and Biophysics, University of Tehran, Tehran, Iran; ^10^Biology Department, Faculty of Sciences-I, Lebanese University, Beirut, Lebanon

**Keywords:** multiple sclerosis, melatonin, pyruvate dehydrogenase kinase, pyruvate dehydrogenase complex, myelin

## Abstract

Metabolic disturbances have been implicated in demyelinating diseases including multiple sclerosis (MS). Melatonin, a naturally occurring hormone, has emerged as a potent neuroprotective candidate to reduce myelin loss and improve MS outcomes. In this study, we evaluated the effect of melatonin, at both physiological and pharmacological doses, on oligodendrocytes metabolism in an experimental autoimmune encephalomyelitis (EAE) mouse model of MS. Results showed that melatonin decreased neurological disability scores and enhanced remyelination, significantly increasing myelin protein levels including MBP, MOG, and MOBP. In addition, melatonin attenuated inflammation by reducing pro-inflammatory cytokines (IL-1β and TNF-α) and increasing anti-inflammatory cytokines (IL-4 and IL-10). Moreover, melatonin significantly increased brain concentrations of lactate, N-acetylaspartate (NAA), and 3-hydroxy-3-methylglutaryl-coenzyme-A reductase (HMGCR). Pyruvate dehydrogenase kinase-4 (PDK-4) mRNA and protein expression levels were also increased in melatonin-treated, compared to untreated EAE mice. However, melatonin significantly inhibited active and total pyruvate dehydrogenase complex (PDC), an enzyme under the control of PDK4. In summary, although PDC activity was reduced by melatonin, it caused a reduction in inflammatory mediators while stimulating oligodendrogenesis, suggesting that oligodendrocytes are forced to use an alternative pathway to synthesize fatty acids for remyelination. We propose that combining melatonin and PDK inhibitors may provide greater benefits for MS patients than the use of melatonin therapy alone.

## Introduction

Multiple sclerosis (MS) is a complex disease characterized by inflammation and demyelination in the central nervous system (CNS), affecting approximately 2.5 million people worldwide (Hagemeier et al., 2012). Although some immunological, genetic, and environmental factors have been identified and associated with MS, the molecular and biochemical mechanisms underlying this pathology are not fully understood. In fact, MS has been shown to cause neurodegeneration through different mechanisms including oxidative stress, activation of microglia and astrocytes, impairment in energy state and metabolism, and other processes (Mahad et al., 2015; Heidker et al., 2017; Kawachi and Lassmann, 2017). In addition, different kinds of immune cell mediators are involved in the pathogenesis of MS. Indeed, through diverse collections of T cells, the entire inflammatory infiltrate of the demyelinated area was shown to be eliminated by down-regulation of proinflammatory cytokines and up-regulation of suppressive cytokines in an animal model of MS (Brocke et al., 1996; Steinman, 1996). Current therapies are using medications to suppress immunological attacks on myelin, without suppressing the entire immune system (Steinman, 1996). Moreover, metabolic alterations in MS have been investigated in relation to the multiple pathophysiological processes linking mitochondrial function, myelin, and inflammation (Kalman et al., 2007). Among metabolic pathways, lipid synthesis is a critical process for remyelination since lipids and cholesterol are key components of myelin and their increased synthesis has been related to the therapeutic mechanism of MS medications (Sedel et al., 2016). Fatty acid (FA) synthesis and/or lipid availability are considered to be rate-limiting steps for myelin synthesis, as inhibition of cholesterol synthesis caused hypomyelination and delayed myelination in mice (Saher et al., 2005). In order to be clinically effective, therapeutic agents of MS need to have broad neuroprotective effects targeting these multiple mechanisms.

Recently, melatonin, a naturally occurring hormone secreted by the pineal gland, has been introduced as a promising neuroprotective candidate to improve MS outcomes (Sandyk, 1993; Kang et al., 2001; Melamud et al., 2012; Alvarez-Sanchez et al., 2015; Chen et al., 2016). Melatonin has been reported to exert its beneficial effects by reducing oxidative stress (Tan et al., 2007), modulating energy metabolism (Leon et al., 2004), and attenuating apoptosis and inflammatory response (Altun and Ugur-Altun, 2007). Importantly, melatonin is selectively taken up by mitochondrial membranes (Srinivasan et al., 2011), where it seems to accumulate in high concentrations (Martin et al., 2000), preserving their integrity and improving their function. Therefore, studying the effects of melatonin on myelination and mitochondrial metabolism in MS represents an important area of investigation.

It has been previously shown that melatonin protects against experimental autoimmune encephalomyelitis (EAE) by controlling peripheral and central T effector/regulatory responses (Alvarez-Sanchez et al., 2015) or through suppression of intercellular adhesion molecule-1 (Kang et al., 2001) in adult mouse or rat models of EAE. In contrast, we recently reported a negative impact for melatonin on EAE recovery of young rats, suggesting a relationship between age and the development of EAE (Ghareghani et al., 2017a). Indeed, administration of 10 mg/kg/day of melatonin to 5–6 weeks old rats caused an increase in pro-inflammatory cytokine levels and activation of astrocytes, which seemed to delay the remyelination process by increasing the accumulation of lactate. Our previous studies on the mouse EAE model showed that exogenous melatonin reduced the oxidative stress while increasing the antioxidant enzymatic activity resulting into promoting oligodendrogenesis (Ghareghani et al., 2018b). It should be noted that we recently showed, for the first time, that melatonin increased oligodendrocyte differentiation from neural stem cells *in vitro* (Ghareghani et al., 2017b). Interestingly, melatonin was previously shown to reduce the levels of microglial activation and oligodendroglial maturation in an injury model of the White Matter (Olivier et al., 2009).

Furthermore, the beneficial role of melatonin at the clinical level was confirmed in Relapsing Remitting MS by attenuating the levels of pro-inflammatory cytokines and oxidative stress (Sánchez-López et al., 2008). In addition, studies on demyelinated diseases showed that melatonin exerts its neuroprotective effects through activation of the Nrf2/ARE pathway, a key defense regulator against oxidative stress in the body (Long et al., 2018).

In this study, in order to further investigate the effect of melatonin on energy metabolism, we used the EAE mouse model to assess any alterations in mitochondrial function and metabolic enzymes as well as the expression of demyelination and inflammatory mediators. Our results demonstrated that pathological findings were substantially improved by melatonin therapy, which was found to modulate cerebral metabolism and to enhance the remyelination process.

## Materials and Methods

### Experimental Animals

Adult female C57BL/6 mice (6–8 weeks old, 20–25 g), were purchased from Iran Pasteur Institute (Pasteur’s Institute, Tehran, Iran). The Institutional Animal Care and Use Committee (IACUC) of Tehran University approved all experimental procedures in this study. Mice were maintained and housed under pathogen-free conditions with constant temperature and humidity control at the Animal Breeding Center under a 14/10 light/dark cycle. Animal experimental procedures were carried out in accordance with the guidelines of the Iranian Agriculture Ministry, which conforms to the provisions of the Declaration of Helsinki (as revised in Brazil in 2013), and of the European Communities Council Directive (86/609/EEC).

### EAE Induction

Mice were immunized with myelin oligodendrocyte glycoprotein peptide (MOG)_35-55_ (MEVGWYRSPFSRVVHLYRNGK) purchased from Hooke Laboratories (Lawrence, MA, United States). MOG_35-55_ was emulsified in complete Freund’s adjuvant (CFA, Sigma Aldrich), enriched Mycobacterium tuberculosis bacteria. Briefly, on day 1, each mouse was anesthetized with isoflurane (Abbott Labs, United States), injected with 10 μl of MOG emulsion subcutaneously over the flank and then injected intraperitoneally with 200 ng of pertussis toxin (PTX) (Hooke Laboratories, Lawrence, MA, United States), diluted in sterile PBS. On day 3, a second 200 ng booster PTX injection was given.

### Clinical Evaluation of EAE Mice

Mice were evaluated and scored for clinical signs of the disease by at least 2 investigators from days 7 to 30 post-immunization using a 0–5 point scale (Nashold et al., 2013), as follows: 0 = no clinical disease; 0.5, partial tail paralysis; 1.0, complete tail paralysis or limp tail; 1.5, complete tail paralysis and partial paralysis one hind limb; 2.0, complete tail paralysis and partial paralysis of both hind limbs; 2.5, partial paralysis of one hind limb and complete paralysis of one hind limb; 3.0, paralysis of both hind limbs without forelimb weakness; 4.0, hind limbs and one forelimb paralysis; 5.0, moribund/dead. Mice were also weighed daily after immunization.

### Treatment of Animals

**FIGURE 1:**
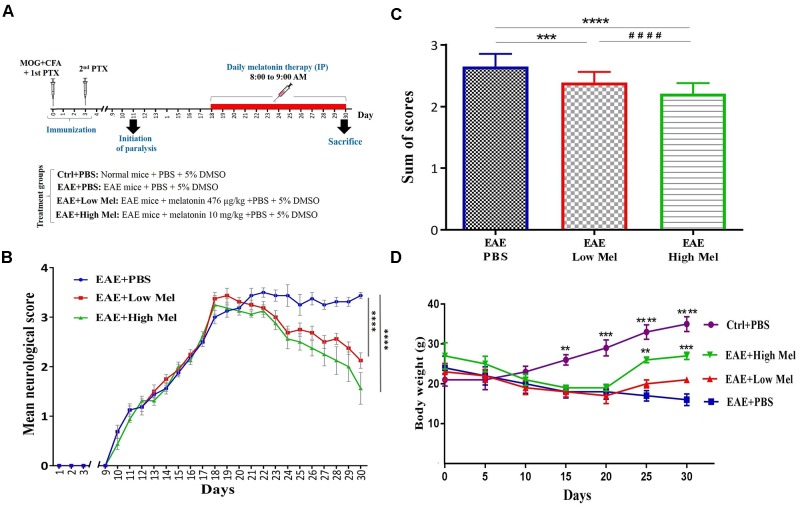
Melatonin ameliorates the clinical scores of EAE. **(A)** Schematic representation of the experimental procedures. EAE was induced in C57BL6 mice (day 0) which were treated at day 18 with PBS, low or high doses of melatonin until day 29. Daily clinical scores were then measured, which continued until day 30. **(B)** Neurological disability analysis displayed an amelioration in EAE disease severity in melatonin treated mice compared to PBS treated EAE mice. **(C)** Cumulative neurological disability was significantly lower in mice treated with physiological (476 μg/kg/day) or pharmacological (10 mg/kg/day) doses of melatonin, compared to PBS treated EAE mice. **(D)** The mean body weight was recorded at 5 days interval. Melatonin treated mice significantly gained body weight at days 25 and 30, in comparison to vehicle. Values are expressed as the Mean ± SEM. Each group included 8 mice (*n* = 8). Statistical analysis was performed by two-way analysis of variance (ANOVA) followed by Tukey’s test. Significance is indicated by ^∗∗^*p* < 0.01, ^∗∗∗^*p* < 0.001, and ^∗∗∗∗^*p* < 0.0001 vs. vehicle and ^####^*p* < 0.0001 vs. EAE-low melatonin.

Mice were randomly divided into 4 groups of: (A) Control phosphate buffered saline (PBS)-treated mice (Ctrl) (*n* = 8); (B) Vehicle PBS-treated EAE mice (Vehicle) (*n* = 8), (C) low-dose melatonin treated EAE mice (Low Mel) at physiological levels of 476 μg/kg/day (*n* = 8), and (D) high-dose melatonin treated EAE mice (High Mel) at pharmacological levels of 10 mg/kg/day (*n* = 8). To be clinically relevant, treatment was given intraperitoneally (i.p.) for 13 consecutive days (from days 18 to 30), starting on the day of clinical symptom onset (score ≥ 3), until sacrifice at day 30. All treatments were done between 8:00 and 9:00 AM, when melatonin level is at its lowest. On the other hand, mice were sacrificed at day 30, 11–12 h after the last treatment, before the onset of darkness. Melatonin (Sigma-Aldrich, United States) was freshly prepared by dissolving it in PBS and 5% dimethyl sulfoxide (DMSO) then administrated at the doses described above. Physiological and pharmacological doses of Melatonin were chosen based on previous studies by Hamdi (1998) and Ghareghani et al. (2017a), respectively. Control, vehicle (EAE mice) and experimental groups all received the same percentage of 5% DMSO. The experimental procedures are schematized in Figure 1A.

### Sample Preparation

Mice were anesthetized and then sacrificed by cervical dislocation and brain tissues quickly excised, immediately frozen on dry ice and stored at -80°C until further tests performed.

### Quantifying Cytokines by Enzyme-Linked Immunosorbent Assay (ELISA)

The brain levels of pro-inflammatory cytokines IL-1β and TNF-α and anti-inflammatory cytokines IL-4 and IL-10 were measured using ELISA kit in accordance with the manufacturer’s instructions (R&D Systems, Minneapolis, MN and Abcam, United States).

### High Performance Liquid Chromatography (HPLC)

Brain homogenate samples were centrifuged at 40,000 × *g* for 15 min, supernatants placed in an ice bath and neutralized to pH 4–5 with potassium hydroxide (KOH), and then pellets were weighed for analysis. Samples were centrifuged for another 15 min at 40,000 × *g* for 15 min to sediment the precipitant, potassium perchlorate (KClO4), which formed after neutralization with KOH. Supernatants were retained and filtered (0.2 μm) before lyophilization. The chromatographic measurements of brain lactate and NAA were carried out with a KNAUER smartline High Performance Liquid Chromatography (HPLC) system equipped with micro vacuum degasser, LPG system, UV-VIS Detector (2550 was set at 220 nm) and a MZ ODS-C18 (250 mm × 4.6 mm, 5 μm) column. The chromatographic calculations were performed using an EZCHROM elite system. Determination of lactate and NAA were performed by HPLC as previously described (Kehr, 1999; Shannon et al., 2016). The accuracy of extraction and determination of lactate and NAA in the brain were investigated using standard addition method.

### Western Blotting

Brain samples were homogenized on ice and lysed in a lysis buffer containing 50 mM Tris–HCl (pH 7.5), 150 mM NaCl, 0.5% deoxycholic acid, 1% Nonidet P40, 0.1% SDS, 1 mM PMSF, and 100 mg/ml leupeptin. Protein content was measured using a Bio-Rad colorimetric protein assay kit (Bio-Rad, United States). An equal amount of total protein (40 μg) was resolved on 8–15% sodium dodecyl sulfate polyacrylamide gel and then transferred onto a nitrocellulose membrane. The membranes were blocked for 1 h in 5% skim milk solution, and then incubated with primary antibodies against myelin oligodendrocyte glycoprotein (MOG; 1:500, Abcam, United States), myelin-associated oligodendrocytic basic protein (MOBP; 1:500, Abcam), myelin basic protein (MBP; 1:500, Abcam), Pyruvate Dehydrogenase Kinase isoform 4 (PDK4, 1:600, Abcam), and β-actin (1:1000, Santa Cruz Biotechnology Inc., United States) for an overnight on shaker at 4°C. After washing, horseradish peroxidase-conjugated species appropriate secondary antibodies were incubated at room temperature.

Immunoreactive proteins were detected with an enhanced chemiluminescence Western blotting detection system. The relative density of the protein bands was scanned by densitometry using MyImage (SLB, Seoul, Korea), and quantified by image analysis software for gel documentation (LabWorks Software Version 3.0, UVP Inc., United States).

### Real-Time PCR

Total RNA was isolated from brain homogenates using Tri-Reagent (Sigma-Aldrich, the Netherlands), according to the manufacturer’s protocol. Then cDNA was synthesized with High-Capacity cDNA Reverse Transcription kit using random primers (Applied Biosystems, United States). Quantitative real-time PCR (qRT-PCR) was performed by the StepOne Real-Time PCR system (Applied Biosystems). Real-time PCR was carried out with RealQ Plus 2x Master Mix Green (Ampliqon, Denmark) according to manufacturer’s instructions. Primer sequences used were the following: PDK4 F, CCGCTTAGTGAACACTCCTTC, and R, TCTACAAACTCTGACAGGGCTTT; HMGCR F TGATTGGAGTTGGCACCAT, and R, TGGCCAACACTGACATGC. The specificity of PCR products was confirmed by melting curve analysis. The PCR conditions were as follows: initial activation at 95°C for 15 min, then 35 amplification cycles consisting of denaturation at 95°C for 15 s, annealing at 57°C for 30 s, and extension at 72°C for 30 s. The relative changes in gene expression levels were determined by the Comparative CT (ΔΔCT) method. All reactions were performed in triplicate using β-actin as an internal control for normalization.

### PDC Activity Determination

Pyruvate dehydrogenase complex (PDC) exists in two forms: dephosphorylated “active” and phosphorylated “inactive” forms. Inter-conversion between these forms can readily alter the flux through this complex. Hence, both activities were measured because it’s more accurate to evaluate the change in PDC activity. Either active or total PDC activities were measured on ice freeze-thawed homogenates of brains of all groups on day 18, as described previously (Johnson et al., 2001; Pliss et al., 2004, 2013). PDC activity was determined by production of ^14^CO_2_ [^1-14^C] from pyruvate. To preserve the “active” PDC activity, homogenizing buffer contained dichloroacetate (inhibitor of PDH kinases) and sodium fluoride (inhibitor of PDH phosphatases) (both from Santa Cruz Biotechnologies, United States). For measurement of “total” PDC activity, purified recombinant PDH phosphatase 1 was added to freeze-thawed homogenates and incubated for 30 min to dephosphorylate phospho-PDH. PDC activity is expressed asmunits/mg protein.

### Statistical Analysis

Results are presented as means with error bars indicating the standard error of the mean (Mean ± SEM). There was no evidence for significant deviations from normal distribution (*p* > 0.05, Shapiro–Wilk test). RM two-way analysis of variance (ANOVA) and RM one-way ANOVA with the greenhouse-Geisser correction test, followed by Tukey’s multiple comparisons test, were used for Figure 1A,B, respectively. On the other hand, ANOVA followed by Tukey’s multiple comparison test with a single pooled variance was used to analyze all other figures. All statistical tests were two-sided and the level of significance was set at *p* < 0.05. GraphPad Prism software version 6.01 (San Diego, CA, United States) was used to perform statistical analysis. Significance is indicated by ^∗^*p* < 0.05; ^∗∗^*p* < 0.01; ^∗∗∗^*p* < 0.001, and ^∗∗∗∗^*p* < 0.0001.

## Results

### Melatonin Ameliorates Clinical Signs of EAE

To assess whether melatonin ameliorates EAE severity, mice were treated by daily i.p. injections of melatonin at physiological (476 μg/kg/day) or pharmacological doses (10 mg/kg/day), from day 18 post-first immunization until the end of the study at day 30. The disease course in this model exhibits a chronic progressive-relapsing phenotype. When compared to the vehicle group (EAE+PBS), both low and high melatonin treated EAE mice showed a significant reduction (*p* < 0.0001) in the severity of disease, starting at day 23 and which continued until day 30, as assessed by the analysis of clinical symptoms (Figure 1B). The peak mean clinical score in untreated EAE mice was as high as 3.5 at 30 days, which declined to 2.1 and 1.6 in low and high melatonin treated mice, respectively (Figure 1B). Moreover, cumulative neurological disability was significantly lower in mice treated with low or high melatonin, compared to untreated EAE mice (*p* < 0.05 and *p* < 0.01, respectively, Figure 1C). Furthermore, EAE mice showed significant and continuous body weight loss 15 days after immunization, in comparison to control mice (Figure 1D). In contrast, EAE mice treated with pharmacological doses of melatonin showed a significant gain in their body weight at days 25 and 30 (^∗∗^*p* < 0.01, ^∗∗∗^*p* < 0.001, respectively), in comparison to vehicle (EAE+PBS) (Figure 1D). Together, these results suggest that melatonin suppresses the development and progression of EAE.

### Melatonin Attenuates Intracerebral Inflammation

**FIGURE 2:**
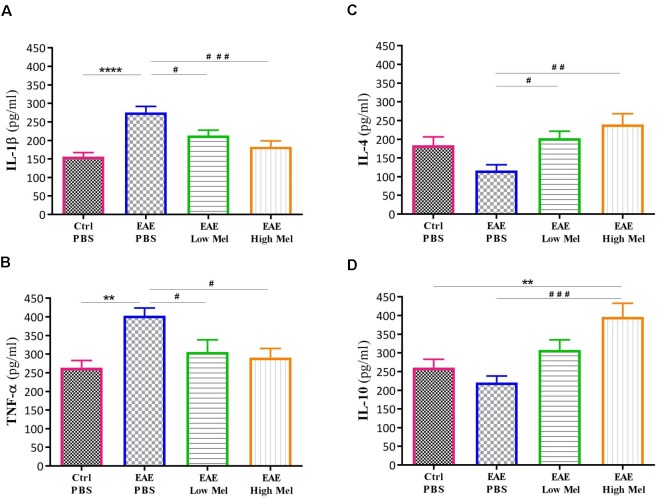
Effect of melatonin on cytokine levels in brain, using ELISA. Levels of pro-inflammatory cytokines **(A)** IL-1β and **(B)** TNF-α. Melatonin significantly suppressed their release. Levels of anti-inflammatory cytokines **(C)** IL-4 and **(D)** IL-10. Melatonin significantly increased the levels of IL-4 and IL-10. Values are expressed as the Mean ± SEM. Each group included 8 mice (*n* = 8). Statistical analysis was performed by two-way analysis of variance (ANOVA) followed by Tukey’s test. Significance is indicated by ^∗∗^*p* < 0.01, ^∗∗∗∗^*p* < 0.0001 vs. control-PBS and ^#^*p* < 0.05, ^##^*p* < 0.01, and ^###^*p* < 0.001 vs. EAE-PBS.

To investigate the role of inflammation in melatonin-induced amelioration of the disease, key cytokines of both pro-inflammatory (IL-1β and TNF-α) and anti-inflammatory (IL-4 and IL-10) pathways were evaluated in non-perfused CNS homogenates (Figure 2). Brain levels of IL-1β and TNF-α were significantly higher in EAE mice at day 30, compared with controls (*p* < 0.0001 and *p* < 0.01, respectively, Figure 2A,B). However, both low- and high-dose melatonin treatment resulted in a substantial decrease in the levels of IL-1β and TNF-α in comparison to the control EAE group (*p* < 0.05, Figure 2A,B).

On the other hand, the levels of IL-4 and IL-10 in untreated EAE mice were slightly, but not significantly, decreased compared with controls. However, IL-4 was significantly increased in low or high melatonin treatment (*p* < 0.05 and *p* < 0.01, respectively, Figure 2C), whereas IL-10 was only increased in high melatonin treated mice, compared to untreated EAE mice (*p* < 0.001, Figure 2D). Our results indicate that the beneficial effects of melatonin in EAE are linked to an inhibition of inflammatory cytokines and an enhanced production of anti-inflammatory cytokines, which tend to be dose-dependent.

### High Melatonin Modulates Cerebral Energy Metabolism and Enhances Mitochondrial Dysfunction

**FIGURE 3:**
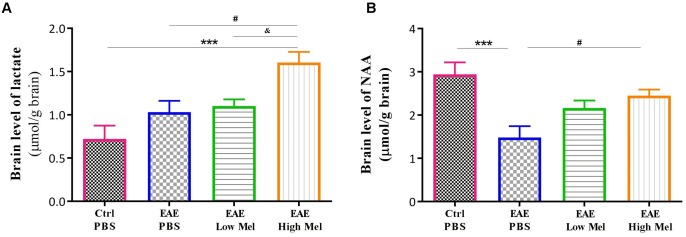
Effects of melatonin on brain lactate and NAA. Levels of brain **(A)** lactate and **(B)** N-acetylaspartate (NAA) were measured by HPLC at day 30 post-first immunization. Only pharmacological doses (High Mel) of melatonin significantly increased brain levels of lactate and NAA, compared to PBS treated EAE mice. Data are expressed as μmol/g brain. Values are expressed as the Mean ± SEM. Each group included 8 mice (*n* = 8). Statistical analysis was performed by two-way analysis of variance (ANOVA) followed by Tukey’s test. Significance is indicated by ^∗∗∗^*p* < 0.001 vs. control-PBS, ^#^*p* < 0.05 vs. EAE-PBS, and & vs. EAE-Low melatonin.

To assess the effect of melatonin on brain metabolism and mitochondrial function, we measured brain lactate and N-acetyl aspartate (NAA). Lactate brain concentrations tended to be higher in EAE mice compared with controls, without reaching statistical significance (Figure 3A). Administration of high-dose, but not low-dose, melatonin resulted in a significant increase in brain lactate concentrations, in comparison to controls (*p* < 0.001) and untreated EAE mice (*p* < 0.05) (Figure 3A). On the other hand, NAA brain concentrations significantly decreased in untreated EAE mice, compared to the control group (*p* < 0.001, Figure 3B). However, high-, but not low-, melatonin treatment significantly increased NAA levels, compared to untreated EAE mice (*p* < 0.05, Figure 3B). Together, these results demonstrate that high-dose melatonin has an effect on cerebral energy metabolism and appears to reverse mitochondrial dysfunction.

### Melatonin Restores HMGCR Gene Expression Levels in the Brain

**FIGURE 4:**
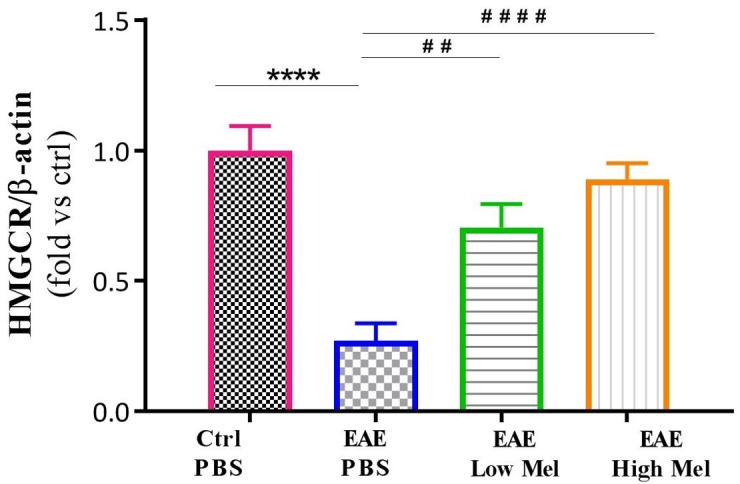
Effect of melatonin on brain HMGCR. mRNA expression levels of 3-hydroxy-3-methylglutaryl-Coenzyme A reductase (HMGCR) were measured in brain homogenates. β-actin was used as an internal control. Quantification of HMGCR expression levels was normalized to controls. Values are expressed as the Mean ± SEM. Each group included 8 mice (*n* = 8). Statistical analysis was performed by two-way analysis of variance (ANOVA) followed by Tukey’s test. Significance is indicated by ^∗∗∗∗^*p* < 0.0001 vs. Control-PBS and ^##^*p* < 0.01, and ^####^*p* < 0.0001 vs. EAE-PBS.

The effect of melatonin treatment was also investigated on a key enzyme in cholesterol biosynthesis, namely 3-hydroxy-3-methylglutaryl-coenzyme-A reductase (HMGCR). Results indicated that HMGCR mRNA levels were significantly reduced in PBS treated EAE mice, compared to the control group (*p* < 0.0001, Figure 4). In contrast, low or high melatonin therapy significantly increased HMGCR levels, compared with untreated EAE mice, in a dose-dependent manner (*p* < 0.01 and *p* < 0.0001, respectively, Figure 4).

### Melatonin Upregulates MBP, MOG, and MOBP Oligodendrocytic Markers

**FIGURE 5:**
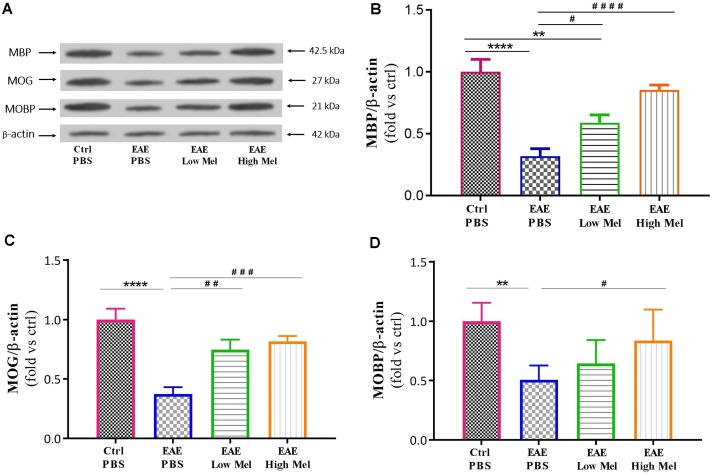
Effect of melatonin on oligodendrocyte markers in brain homogenates. **(A)** Western blot analysis of myelin basic protein (MBP), myelin oligodendrocyte glycoprotein (MOG), and myelin-associated oligodendrocytic basic protein (MOBP) in the brain. All three markers (MOBP, MOG, and MBP) were run alongside the same β-actin as housekeeping control. **(B–D)** Quantitative analysis for MBP, MOG and MOBP proteins, respectively, was performed using LabWorks Software. Melatonin caused a significant increase in the expression of MBP, MOG, and MOBP proteins, in comparison to PBS treated EAE mice. Values are expressed as the Mean ± SEM. Each group included 8 mice (*n* = 8). Statistical analysis was performed by one-way analysis of variance (ANOVA) followed by Tukey’s test. Significance is indicated by ^∗∗^*p* < 0.01, ^∗∗∗∗^*p* < 0.0001 vs. control-PBS and ^#^*p* < 0.05, ^##^*p* < 0.01, ^###^*p* < 0.001, and ^####^*p* < 0.0001 vs. EAE-PBS.

To test whether melatonin affects the protein expression levels of oligodendrocytic markers, brain lysates were used to perform Western blot analysis on myelin basic protein (MBP), myelin oligodendrocyte glycoprotein (MOG), and myelin-associated oligodendrocytic basic protein (MOBP). Results showed a significant decrease in protein levels of MBP, MOG, and MOBP in untreated EAE mice, compared with controls (*p* < 0.0001, *p* < 0.0001, and *p* < 0.01, respectively) (Figure 5A–C). However, high-dose melatonin caused a significant increase in protein expression levels of MBP, MOG, and MOBP, compared to untreated EAE group (^∗∗∗^*p* < 0.001, ^∗∗∗^*p* < 0.001, and ^∗^*p* < 0.05, respectively) whereas low melatonin increased only MBP and MOG protein levels (^∗^*p* < 0.05 and ^∗∗^*p* < 0.01, respectively, Figure 5). Therefore, administration of melatonin appears to induce the expression of oligodendrocytic proteins in EAE mice.

### Melatonin Upregulates PDK4

**FIGURE 6:**
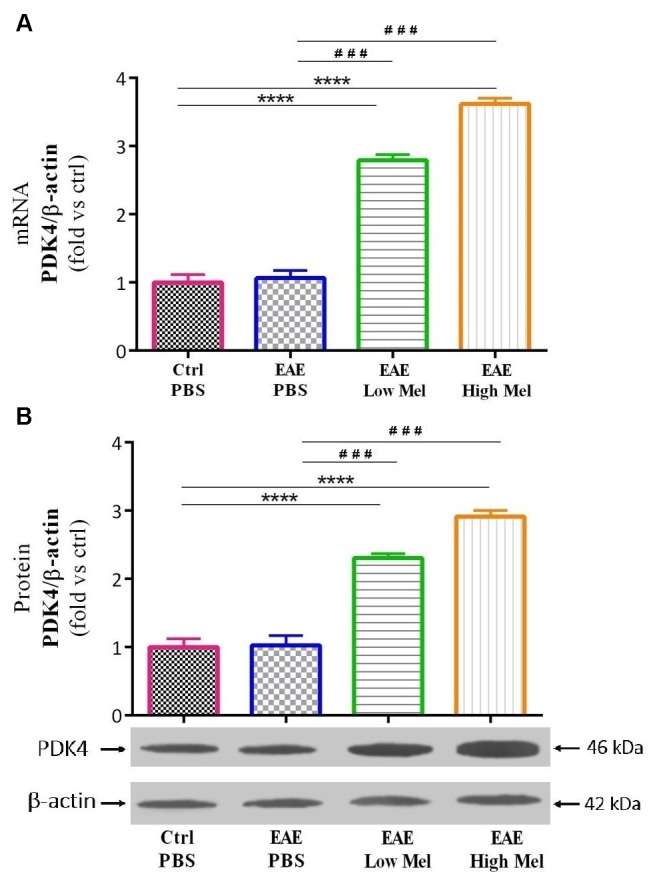
The effects of melatonin on PDK4 levels in brain homogenate. **(A)** mRNA expression levels of PDK4 assessed by real time-PCR. **(B)** Protein expression levels of PDK4 assessed by western-blot. β-actin was used as an internal control for normalization for both real time-PCR and western blot. Quantification PDK4 expression levels was normalized to controls. Values are expressed as the Mean ± SEM. Each group included 8 mice (*n* = 8). Statistical analysis was performed by one-way analysis of variance (ANOVA) followed by Tukey’s test. Significance is indicated by ^∗∗∗^*p* < 0.001 and ^∗∗∗∗^*p* < 0.001 vs. control-PBS and ^###^*p* < 0.001 vs. EAE-PBS.

The effect of melatonin on PDK4 mRNA expression levels was then investigated by quantitative real time-PCR. Induction of EAE mice did not affect PDK4 mRNA expression levels compared with controls at day 30 (Figure 6A). However, low- and high-dose melatonin treatment resulted in a significant∼ 2.8- and 3.6-fold increase in PDK4 mRNA levels, compared with untreated EAE mice (*p* < 0.0001; Figure 6A). Consistent with transcriptional data, Western-blot analysis showed ∼2.3- and ∼2.9-fold increase in PDK4 protein levels, compared with untreated EAE mice (*p* < 0.001; Figure 6B). Thus, these results indicated that melatonin is not only involved but also modulates brain glucose metabolism.

### Melatonin Suppresses the Activity of PDC Enzyme

**FIGURE 7:**
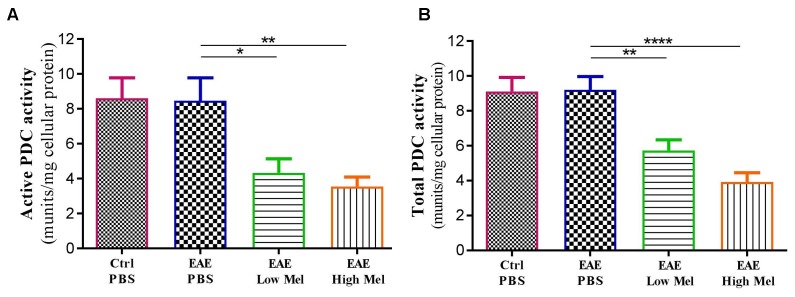
The effects of melatonin on PDC activities in brain homogenates. The change in the activity of PDC enzyme was measured either at the **(A)** “active” or **(B)** “total” forms of PDC. Melatonin significantly decreased the activities of both PDC forms, in comparison to PBS treated EAE mice. Values are expressed as the Mean ± SEM. Each group included 8 mice (*n* = 8). Statistical analysis was performed by one-way analysis of variance (ANOVA) followed by Tukey’s test. Significance is indicated by ^∗^*p* < 0.05, ^∗∗^*p* < 0.01, and ^∗∗∗∗^*p* < 0.0001.

Both “Active” and “Total” PDC activities were then assayed in brain homogenates from mice at day 30 (Figure 7). PDC activity was found to be similar between untreated EAE and control mice. However, melatonin treatment caused a significant dose-dependent reduction in both “active” and “total” forms of PDC enzyme, compared with untreated EAE mice (Figure 7A,B).

## Discussion

**FIGURE 8:**
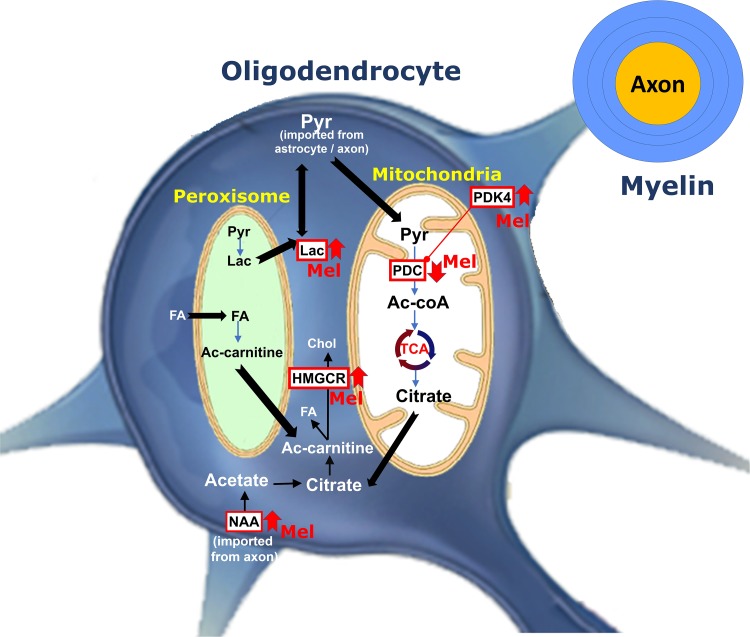
Schematic representation for the role of melatonin on oligodendrocyte metabolism during remyelination. Pyruvate imported into myelin is converted within mitochondria by PDC to produce acetyl-CoA, which generates citrate by TCA. Citrate is subsequently used to produce fatty acid (FA) and cholesterol in the cytosol. PDK4 acts as one of the regulators of PDC activity. Melatonin increases PDK4 expression levels and cause suppression of PDC by its phosphorylation. This results into pyruvate being transferred to peroxisome where FA breakdown occurs to provide redox balance and acetyl-CoA. On the other hand, melatonin increases the levels of NAA. Axonal NAAs are transferred into oligodendrocytes and are converted to acetate and then acetyl-CoA, a substrate for FA and cholesterol synthesis. Finally, melatonin upregulates the expression of HMGCR, a key factor in cholesterol synthesis. (PDC, Pyruvate dehydrogenase complex; PDK4, pyruvate dehydrogenase kinases 4; Ac-CoA, Acetyl-CoA; Ac-carnitine, Acetyl-carnitine; HMGCR, 3-hydroxy-3-methylglutaryl-Coenzyme A reductase; TCA, tricarboxylic acid cycle; Chol, Cholesterol; Pyr, Pyruvate; NAA, N-acetylaspartate, Lac, lactate, FA, Fatty acid, Mel, melatonin).

In the current study, we demonstrated that administration of melatonin at physiological and pharmacological doses ameliorates the severity of EAE by attenuating neuroinflammation, increasing the expression levels of mature oligodendrocytic markers and modulating cerebral energy metabolism, as summarized in Figure 8.

Melatonin was previously shown to be inversely correlated with the severity of MS and its relapse (Sandyk, 1993; Melamud et al., 2012). Several studies have indicated a beneficial role for melatonin in attenuating neuroinflammation and stimulating remyelination in adult EAE mouse and rat models of MS (Kang et al., 2001; Alvarez-Sanchez et al., 2015; Chen et al., 2016). Melatonin is also currently used in an open clinical trial in patients of MS (ClinicalTrials.gov identifier NCT03498131). In this study, the effects of melatonin therapy on the mechanisms of molecular immune polarization were investigated by measuring pro-inflammatory (TNF and IL-1β) and anti-inflammatory (IL-4 and IL-10) cytokine levels in the mouse brain, previously shown to be linked to EAE disability and tissue destruction (Batoulis et al., 2010; Bernardes et al., 2013). Our data in this study confirmed our previous results that pharmacological doses of melatonin significantly recovered the decreased expression levels of IL-4 and IL10 in PBS-treated EAE mice while reducing the elevated levels of TNF and IL-1β (Ghareghani et al., 2018a,b). Moreover, this study demonstrated that MBP and MOG, which are myelin-associated proteins localized on the surface of oligodendrocytes, showed marked reduction in EAE mice compared to controls. However, physiological or pharmacological doses of melatonin significantly increased the expression levels of these proteins, in comparison to the PBS-treated EAE mice. Furthermore, MOBP, a major component of myelin in the periaxonal membrane and which modulates the radial growth of axon and myelin (Yool et al., 2002), has been previously reported to decrease in EAE mice (Carmody et al., 2002). Interestingly, our data in this study demonstrated that pharmacological doses of melatonin could increase MOBP expression, in comparison to EAE mice. While most previous research focused on the effect of melatonin on inflammatory mediators and its anti-oxidant and anti-apoptotic actions (Husson et al., 2002; Kilic et al., 2004; Leon et al., 2004; Tan et al., 2007; Welin et al., 2007; Gressens et al., 2008), the present study investigated its effect on metabolic pathways involved in cerebral metabolism and myelination. Our results showed, for the first time, that melatonin treatment increased PDK4 levels and suppressed PDC activity in an EAE mouse model of MS.

It has been reported that keeping PDC in an active state decreases lactate accumulation in body tissues (Parikh et al., 2009). Recently, we showed that administration of melatonin leads to an increase in serum lactate levels in young EAE Lewis rats (Ghareghani et al., 2017a). In the current study on EAE mouse model, melatonin therapy caused an increase in brain lactate levels, while PDC activity was suppressed. This decrease in PDC activity could explain the mechanism by which lactate increases in response to melatonin. In fact, when coupled, oligodendrocytes and astrocytes take up glucose, lactate or pyruvate from peripheral blood vessels to generate metabolites to myelin-forming oligodendrocytes (Tress et al., 2012) as well as to provide energy to neurons in the form of lactate (Hirrlinger and Dringen, 2010). In fact, glucose needs to be converted, through glycolysis pathway, to pyruvate or lactate in order to provide metabolic support for axonal functions (Funfschilling et al., 2012; Lee et al., 2012). On the other hand, lactate, that has been fluxed from blood or astrocytes, is employed by oligodendrocytes as a main substrate to generate ATP and FA, which are required for myelin synthesis (Rinholm and Bergersen, 2014). However, pyruvate, obtained from lactate and glucose conversion or that has been fluxed from blood, needs first to be converted to acetyl-CoA, through the PDC enzyme, in order to produce metabolites for FA synthesis (Fransen et al., 2017).

Importantly, the activity of PDC is inhibited through phosphorylation by PDK’s, especially isoform 4 (PDK4) (Jha et al., 2012). To date, only one study, using microarray analysis, has shown that melatonin administration increased PDK4 mRNA expression levels in mouse CNS (Sharman et al., 2007). In the current study, PDK4 mRNA and protein levels were found to be upregulated in the brain of melatonin treated EAE mice, associated with a reduction in PDC activity. Surprisingly, the suppression of PDC activity following melatonin therapy coincided with an increase in lactate levels, suggesting that the inability to convert pyruvate, as well as lactate, to acetyl-CoA leads to the accumulation of lactate in the CNS and peripheral blood. In agreement, we have recently demonstrated a significant accumulation of serum lactate levels following melatonin therapy (Ghareghani et al., 2017a). On the other hand, remyelination by oligodendrocytes needs FA synthesis from pyruvate that has been imported to mitochondrial matrix whereas myelin maintenance, after myelination, needs FA breakdown which occurs mainly within the peroxisomes by β-oxidation pathway (Rinholm and Bergersen, 2014). Indeed, the latter is a multistep process involving enzymes that are different from those in mitochondria. In fact, FAs first enter the cell via FA protein transporters (including FAT/CD36) present on the cell surface (Lopaschuk et al., 2010). Although the end products of peroxisomal and mitochondrial β-oxidation are different, however, peroxisomal β-oxidation can produce acetyl-CoA from long chain FAs. Indeed, FAs are converted to acetyl-carnitine which is then exported to cytosol where it can be converted to acetyl-CoA, to be used again as a substrate in FA and cholesterol synthesis (Wanders et al., 2015). Acetyl-CoA produced from β-oxidation cannot be converted to lactate, however, it has been suggested that pyruvate imported into the peroxisomes could yield lactate (McClelland et al., 2003). Considering that inhibition of FA synthesis by the PDC-dependent pathway leads to accumulation of pyruvate, it seems that this elevated level of pyruvate by PDC suppression increases peroxisomal β-oxidation. In accordance, administration of pyruvate was shown to increase peroxisomal β-oxidation, which also needs NAD+ originating from the conversion of pyruvate to lactate (McClelland et al., 2003). Therefore, lactate accumulation is obtained not only through the suppression of pyruvate consumption by PDC, which in turn reduces lactate conversion to pyruvate through a negative feedback mechanism, but also through peroxisomes (Figure 8). Taken together, these observations demonstrate that melatonin affects the metabolic pathway involved in remyelination by increasing the myelination and attenuating the severity of EAE. Additional studies will be required to investigate the link between melatonin and FA synthesis, and to further assess their effects on myelination.

Cholesterol biosynthesis occurs principally (∼95%) in the brain and is a main constituent of myelin sheath (Muse et al., 2001; Bjorkhem and Meaney, 2004; Dietschy, 2009). Therefore, we investigated 3-hydroxy-3-methylglutaryl-coenzyme-A reductase (HMGCR), a key enzyme in cholesterol synthesis. Previous studies have shown that HMGCR is decreased at the peak of acute EAE disease (Lavrnja et al., 2017). However, we demonstrated that melatonin therapy significantly increased HMGCR levels. This observation suggests that cholesterol synthesis is not suppressed by melatonin but rather stimulated by it.

On the other hand, N-acetyl-aspartate (NAA), a nervous system-specific metabolite whose role has not been fully elucidated, appears to be a key link in biochemical features of CNS metabolism (Moffett et al., 2007) and to be involved in myelination and remyelination. Some studies suggested that neuronal NAA can be exported to oligodendrocytes where it is metabolized to form acetate and then acetyl-CoA, which are preferentially integrated into myelin (Hagenfeldt et al., 1987; Burri et al., 1991; Chakraborty et al., 2001). Previous studies confirmed that brain NAA levels are decreased in MS and that NAA precedes neuronal atrophy, indicating that mitochondrial dysfunction may precede neurodegeneration (Inglese et al., 2004; Cader et al., 2007). Moreover, NAA reduction has been correlated with disease progression and neurological decline in MS patients (Fu et al., 1998; Signoretti et al., 2001). In our study, high melatonin treatment reversed the decreases of NAA levels. Our results give further support to the hypothesis that melatonin enhances mitochondrial energy production and, possibly, remyelination. In accordance with our findings, partial recovery of NAA levels has also been reported in patients treated with interferon beta-1b (Narayanan et al., 2001), fluoxetine (Mostert et al., 2006), or glatiramer acetate (Khan et al., 2005).

Increasing evidence confirms that melatonin ameliorates EAE severity at different concentrations, albeit, the precise optimal dose for this neuroprotective agent is still undefined. Thus, two different doses were used in this study. The “high – pharmacological” dose has been chosen based on our previous study on EAE rat model (Ghareghani et al., 2017a) whereas the “low – physiological” dose was used according to the previous work of Hamdi (1998).

The number of mitochondria increase in demyelinated axons of acute MS (Mahad et al., 2008), thus, the change of mitochondria numbers in untreated and melatonin treated mice remains to be determined. A limitation in the current study is that we used brain lysates which do not allow to determine the origin of the cells responsible for the measured factors. Further studies are needed to investigate these markers in specific cells including astrocytes, neurons, and oligodendrocytes. Moreover, to reach a precise conclusion on melatonin therapy, it is highly recommended to take into account the time of melatonin administration (between 17:00 and 19:00 in this study), the time of sampling (between 10:00 and 12:00 AM), the type of EAE model, the length and dose of melatonin therapy, the route of melatonin administration (i.p. in this study), the age and weight of mice or rats, and finally the protocol of EAE induction.

## Conclusion

In conclusion, in the normal process of FA synthesis in remyelination, cells need acetyl-CoA as substrate which is obtained from pyruvate through the action of PDC (Figure 8). The latter is itself under the control of PDK4. However, in the current study, we observed that although melatonin suppressed the activity of PDC, a drawback of melatonin therapy, it still ameliorated EAE severity, both at physiological and pharmacological doses of melatonin. Indeed, melatonin caused a reduction in inflammatory mediators while stimulating oligodendrogenesis, suggesting that oligodendrocytes are forced to use an alternative pathway to synthesize FA’s for remyelination. This alternative pathway seems to be slower than the main PDC pathway of FA synthesis. Both the source of the alternative substrate and the mechanistic regulators for the substitute FA synthesis pathway are still unknown. Therefore, in order to increase the efficiency of melatonin therapy in EAE, MS or demyelination disorders, we suggest a new treatment strategy through the activation of PDC. This hypothesis would need further experimental and clinical studies by investigating, for instance, the synergistic effect of melatonin and a PDK inhibitor on EAE or MS.

## Data Availability

The datasets generated for this study are available on request to the corresponding author.

## Author Contributions

MG, SG, and KZ designed the study and interpreted results. MG, LS, YJ, NF, HS, AG, SM, and DA performed the experiments, collected the data, and helped in the analysis. MG and KZ wrote the manuscript. All authors have read, critically revised and approved the final manuscript before submission.

## Conflict of Interest Statement

The authors declare that the research was conducted in the absence of any commercial or financial relationships that could be construed as a potential conflict of interest.

## References

[B1] Altun A. Ugur-Altun B. (2007). Melatonin: therapeutic and clinical utilization. *Int. J. Clin. Pract.* 61 835–845. 10.1111/j.1742-1241.2006.01191.x 17298593

[B2] Alvarez-Sanchez N. Cruz-Chamorro I. Lopez-Gonzalez A. Utrilla J. C. Fernandez-Santos J. M. Martinez-Lopez A. (2015). Melatonin controls experimental autoimmune encephalomyelitis by altering the T effector/regulatory balance. *Brain Behav. Immun.* 50 101–114. 10.1016/j.bbi.2015.06.021 26130320

[B3] Batoulis H. Addicks K. Kuerten S. (2010). Emerging concepts in autoimmune encephalomyelitis beyond the Cd4/Th1 paradigm. *Ann. Anat.* 192 179–193. 10.1016/j.aanat.2010.06.006 20692821

[B4] Bernardes D. Oliveira-Lima O. C. Silva T. V. Faraco C. C. Leite H. R. Juliano M. A. (2013). Differential brain and spinal cord cytokine and Bdnf levels in experimental autoimmune encephalomyelitis are modulated by prior and regular exercise. *J. Neuroimmunol.* 264 24–34. 10.1016/j.jneuroim.2013.08.014 24054000

[B5] Bjorkhem I. Meaney S. (2004). Brain cholesterol: long secret life behind a barrier. *Arterioscler. Thromb. Vasc. Biol.* 24 806–815. 10.1161/01.ATV.0000120374.59826.1b 14764421

[B6] Brocke S. Gijbels K. Allegretta M. Ferber I. Piercy C. Blankenstein T. (1996). Treatment of experimental encephalomyelitis with a peptide analogue of myelin basic protein. *Nature* 379 343–346. 10.1038/379343a0 8552189

[B7] Burri R. Steffen C. Herschkowitz N. (1991). N-acetyl-L-aspartate is a major source of acetyl groups for lipid synthesis during rat brain development. *Dev. Neurosci.* 13 403–411. 10.1159/000112191 1809557

[B8] Cader S. Johansen-Berg H. Wylezinska M. Palace J. Behrens T. E. Smith S. (2007). Discordant white matter N-acetylasparate and diffusion Mri measures suggest that chronic metabolic dysfunction contributes to axonal pathology in multiple sclerosis. *Neuroimage* 36 19–27. 10.1016/j.neuroimage.2007.02.036 17398118

[B9] Carmody R. J. Hilliard B. Maguschak K. Chodosh L. A. Chen Y. H. (2002). Genomic scale profiling of autoimmune inflammation in the central nervous system: the nervous response to inflammation. *J. Neuroimmunol.* 133 95–107. 10.1016/S0165-5728(02)00366-1 12446012

[B10] Chakraborty G. Mekala P. Yahya D. Wu G. Ledeen R. W. (2001). Intraneuronal N-acetylaspartate supplies acetyl groups for myelin lipid synthesis: evidence for myelin-associated aspartoacylase. *J. Neurochem.* 78 736–745. 10.1046/j.1471-4159.2001.00456.x 11520894

[B11] Chen S. J. Huang S. H. Chen J. W. Wang K. C. Yang Y. R. Liu P. F. (2016). Melatonin enhances interleukin-10 expression and suppresses chemotaxis to inhibit inflammation in situ and reduce the severity of experimental autoimmune encephalomyelitis. *Int. Immunopharmacol.* 31 169–177. 10.1016/j.intimp.2015.12.020 26735612

[B12] Dietschy J. M. (2009). Central nervous system: cholesterol turnover, brain development and neurodegeneration. *Biol. Chem.* 390 287–293. 10.1515/BC.2009.035 19166320 PMC3066069

[B13] Fransen M. Lismont C. Walton P. (2017). The peroxisome-mitochondria connection: how and why? *Int. J. Mol. Sci.* 18:1126. 10.3390/ijms18061126 28538669 PMC5485950

[B14] Fu L. Matthews P. M. De Stefano N. Worsley K. J. Narayanan S. Francis G. S. (1998). Imaging axonal damage of normal-appearing white matter in multiple sclerosis. *Brain* 121(Pt 1), 103–113. 10.1093/brain/121.1.103 9549491

[B15] Funfschilling U. Supplie L. M. Mahad D. Boretius S. Saab A. S. Edgar J. (2012). Glycolytic oligodendrocytes maintain myelin and long-term axonal integrity. *Nature* 485 517–521. 10.1038/nature11007 22622581 PMC3613737

[B16] Ghareghani M. Dokoohaki S. Ghanbari A. Farhadi N. Zibara K. Khodadoust S. (2017a). Melatonin exacerbates acute experimental autoimmune encephalomyelitis by enhancing the serum levels of lactate: a potential biomarker of multiple sclerosis progression. *Clin. Exp. Pharmacol. Physiol.* 44 52–61. 10.1111/1440-1681.12678 27696474

[B17] Ghareghani M. Sadeghi H. Zibara K. Danaei N. Azari H. Ghanbari A. (2017b). Melatonin increases oligodendrocyte differentiation in cultured neural stem cells. *Cell. Mol. Neurobiol.* 37 1319–1324. 10.1007/s10571-016-0450-4 27987059 PMC11482234

[B18] Ghareghani M. Scavo L. Arnoult D. Zibara K. Farhadi N. (2018a). Melatonin therapy reduces the risk of osteoporosis and normalizes bone formation in multiple sclerosis. *Fundam. Clin. Pharmacol.* 32 181–187. 10.1111/fcp.12337 29193274

[B19] Ghareghani M. Zibara K. Sadeghi H. Farhadi N. (2018b). Spasticity treatment ameliorates the efficacy of melatonin therapy in experimental autoimmune encephalomyelitis (eae) mouse model of multiple sclerosis. *Cell. Mol. Neurobiol.* 38 1145–1151. 10.1007/s10571-018-0580-y 29497878 PMC11481852

[B20] Gressens P. Schwendimann L. Husson I. Sarkozy G. Mocaer E. Vamecq J. (2008). Agomelatine, a melatonin receptor agonist with 5-Ht(2C) receptor antagonist properties, protects the developing murine white matter against excitotoxicity. *Eur. J. Pharmacol.* 588 58–63. 10.1016/j.ejphar.2008.04.016 18466899

[B21] Hagemeier K. Bruck W. Kuhlmann T. (2012). Multiple sclerosis - remyelination failure as a cause of disease progression. *Histol. Histopathol.* 27 277–287. 22237705 10.14670/HH-27.277

[B22] Hagenfeldt L. Bollgren I. Venizelos N. (1987). N-acetylaspartic aciduria due to aspartoacylase deficiency–a new aetiology of childhood leukodystrophy. *J. Inherit. Metab. Dis.* 10 135–141. 10.1007/BF01800038 3116332

[B23] Hamdi A. (1998). Melatonin administration increases the affinity of D2 dopamine receptors in the rat striatum. *Life Sci.* 63 2115–2120. 10.1016/S0024-3205(99)80008-3 9839535

[B24] Heidker R. M. Emerson M. R. Levine S. M. (2017). Metabolic pathways as possible therapeutic targets for progressive multiple sclerosis. *Neural Regen. Res.* 12 1262–1267. 10.4103/1673-5374.213542 28966637 PMC5607817

[B25] Hirrlinger J. Dringen R. (2010). The cytosolic redox state of astrocytes: maintenance, regulation and functional implications for metabolite trafficking. *Brain Res. Rev.* 63 177–188. 10.1016/j.brainresrev.2009.10.003 19883686

[B26] Husson I. Mesples B. Bac P. Vamecq J. Evrard P. Gressens P. (2002). Melatoninergic neuroprotection of the murine periventricular white matter against neonatal excitotoxic challenge. *Ann. Neurol.* 51 82–92. 10.1002/ana.10072 11782987

[B27] Inglese M. Ge Y. Filippi M. Falini A. Grossman R. I. Gonen O. (2004). Indirect evidence for early widespread gray matter involvement in relapsing-remitting multiple sclerosis. *Neuroimage* 21 1825–1829. 10.1016/j.neuroimage.2003.12.008 15050603

[B28] Jha M. K. Jeon S. Suk K. (2012). Pyruvate dehydrogenase kinases in the nervous system: their principal functions in neuronal-glial metabolic interaction and neuro-metabolic disorders. *Curr. Neuropharmacol.* 10 393–403. 10.2174/157015912804143586 23730261 PMC3520047

[B29] Johnson M. T. Mahmood S. Hyatt S. L. Yang H. S. Soloway P. D. Hanson R. W. (2001). Inactivation of the murine pyruvate dehydrogenase (Pdha1) gene and its effect on early embryonic development. *Mol. Genet. Metab.* 74 293–302. 10.1006/mgme.2001.3249 11708858

[B30] Kalman B. Laitinen K. Komoly S. (2007). The involvement of mitochondria in the pathogenesis of multiple sclerosis. *J. Neuroimmunol.* 188 1–12. 10.1016/j.jneuroim.2007.03.020 17493689

[B31] Kang J. C. Ahn M. Kim Y. S. Moon C. Lee Y. Wie M. B. (2001). Melatonin ameliorates autoimmune encephalomyelitis through suppression of intercellular adhesion molecule-1. *J. Vet. Sci.* 2 85–89. 10.4142/jvs.2001.2.2.85 14614276

[B32] Kawachi I. Lassmann H. (2017). Neurodegeneration in multiple sclerosis and neuromyelitis optica. *J. Neurol. Neurosurg. Psychiatry* 88 137–145. 10.1136/jnnp-2016-313300 27671902

[B33] Kehr J. (1999). “Monitoring chemistry of brain microenvironment: biosensors, microdialysis and related techniques,” in *Modern Techniques in Neuroscience Research*, eds Windhorst U. Johansson H. (Heidelberg: Springer), 1149–1198.

[B34] Khan O. Shen Y. Caon C. Bao F. Ching W. Reznar M. (2005). Axonal metabolic recovery and potential neuroprotective effect of glatiramer acetate in relapsing-remitting multiple sclerosis. *Mult. Scler.* 11 646–651. 10.1191/1352458505ms1234oa 16320723

[B35] Kilic E. Kilic U. Reiter R. J. Bassetti C. L. Hermann D. M. (2004). Prophylactic use of melatonin protects against focal cerebral ischemia in mice: role of endothelin converting enzyme-1. *J. Pineal Res.* 37 247–251. 10.1111/j.1600-079X.2004.00162.x 15485550

[B36] Lavrnja I. Smiljanic K. Savic D. Mladenovic-Djordjevic A. Tesovic K. Kanazir S. (2017). Expression profiles of cholesterol metabolism-related genes are altered during development of experimental autoimmune encephalomyelitis in the rat spinal cord. *Sci. Rep.* 7:2702. 10.1038/s41598-017-02638-8 28578430 PMC5457442

[B37] Lee Y. Morrison B. M. Li Y. Lengacher S. Farah M. H. Hoffman P. N. (2012). Oligodendroglia metabolically support axons and contribute to neurodegeneration. *Nature* 487 443–448. 10.1038/nature11314 22801498 PMC3408792

[B38] Leon J. Acuna-Castroviejo D. Sainz R. M. Mayo J. C. Tan D. X. Reiter R. J. (2004). Melatonin and mitochondrial function. *Life Sci.* 75 765–790. 10.1016/j.lfs.2004.03.003 15183071

[B39] Long T. Yang Y. Peng L. Li Z. (2018). Neuroprotective effects of melatonin on experimental allergic encephalomyelitis mice via anti-oxidative stress activity. *J. Mol. Neurosci.* 64 233–241. 10.1007/s12031-017-1022-x 29450696

[B40] Lopaschuk G. D. Ussher J. R. Folmes C. D. Jaswal J. S. Stanley W. C. (2010). Myocardial fatty acid metabolism in health and disease. *Physiol. Rev.* 90 207–258. 10.1152/physrev.00015.2009 20086077

[B41] Mahad D. Ziabreva I. Lassmann H. Turnbull D. (2008). Mitochondrial defects in acute multiple sclerosis lesions. *Brain* 131 1722–1735. 10.1093/brain/awn105 18515320 PMC2442422

[B42] Mahad D. H. Trapp B. D. Lassmann H. (2015). Pathological mechanisms in progressive multiple sclerosis. *Lancet Neurol.* 14 183–193. 10.1016/S1474-4422(14)70256-X 25772897

[B43] Martin M. Macias M. Escames G. Leon J. Acuna-Castroviejo D. (2000). Melatonin but not vitamins C and E maintains glutathione homeostasis in t-butyl hydroperoxide-induced mitochondrial oxidative stress. *FASEB J.* 14 1677–1679. 10.1096/fj.99-0865fje 10973915

[B44] McClelland G. B. Khanna S. González G. F. Butz C. E. Brooks G. A. (2003). Peroxisomal membrane monocarboxylate transporters: evidence for a redox shuttle system? *Biochem. Biophys. Res. Commun.* 304 130–135. 10.1016/S0006-291X(03)00550-3 12705896

[B45] Melamud L. Golan D. Luboshitzky R. Lavi I. Miller A. (2012). Melatonin dysregulation, sleep disturbances and fatigue in multiple sclerosis. *J. Neurol. Sci.* 314 37–40. 10.1016/j.jns.2011.11.003 22137446

[B46] Moffett J. R. Ross B. Arun P. Madhavarao C. N. Namboodiri M. A. A. (2007). N-acetylaspartate in the Cns: from neurodiagnostics to neurobiology. *Prog. Neurobiol.* 81 89–131. 10.1016/j.pneurobio.2006.12.003 17275978 PMC1919520

[B47] Mostert J. P. Sijens P. E. Oudkerk M. De Keyser J. (2006). Fluoxetine increases cerebral white matter Naa/Cr ratio in patients with multiple sclerosis. *Neurosci. Lett.* 402 22–24. 10.1016/j.neulet.2006.03.042 16644118

[B48] Muse E. D. Jurevics H. Toews A. D. Matsushima G. K. Morell P. (2001). Parameters related to lipid metabolism as markers of myelination in mouse brain. *J. Neurochem.* 76 77–86. 10.1046/j.1471-4159.2001.00015.x 11145980

[B49] Narayanan S. De Stefano N. Francis G. S. Arnaoutelis R. Caramanos Z. Collins D. L. (2001). Axonal metabolic recovery in multiple sclerosis patients treated with interferon beta-1b. *J. Neurol.* 248 979–986. 10.1007/s004150170052 11757963

[B50] Nashold F. E. Nelson C. D. Brown L. M. Hayes C. E. (2013). One calcitriol dose transiently increases Helios+ FoxP3+ T cells and ameliorates autoimmune demyelinating disease. *J. Neuroimmunol.* 263 64–74. 10.1016/j.jneuroim.2013.07.016 23968560

[B51] Olivier P. Fontaine R. H. Loron G. Van Steenwinckel J. Biran V. Massonneau V. (2009). Melatonin promotes oligodendroglial maturation of injured white matter in neonatal rats. *PloS One*, 4:e7128. 10.1371/journal.pone.0007128 19771167 PMC2742165

[B52] Parikh S. Saneto R. Falk M. J. Anselm I. Cohen B. H. Haas R. (2009). A modern approach to the treatment of mitochondrial disease. *Curr. Treat. Options Neurol.* 11 414–430. 10.1007/s11940-009-0046-0 19891905 PMC3561461

[B53] Pliss L. Hausknecht K. A. Stachowiak M. K. Dlugos C. A. Richards J. B. Patel M. S. (2013). Cerebral developmental abnormalities in a mouse with systemic Pyruvate dehydrogenase deficiency. *PLoS One* 8:e67473. 10.1371/journal.pone.0067473 23840713 PMC3694023

[B54] Pliss L. Pentney R. J. Johnson M. T. Patel M. S. (2004). Biochemical and structural brain alterations in female mice with cerebral pyruvate dehydrogenase deficiency. *J. Neurochem.* 91 1082–1091. 10.1111/j.1471-4159.2004.02790.x 15569252

[B55] Rinholm J. E. Bergersen L. H. (2014). White matter lactate–does it matter? *Neuroscience* 276 109–116. 10.1016/j.neuroscience.2013.10.002 24125892

[B56] Saher G. Brugger B. Lappe-Siefke C. Mobius W. Tozawa R. Wehr M. C. (2005). High cholesterol level is essential for myelin membrane growth. *Nat. Neurosci.* 8 468–475. 10.1038/nn1426 15793579

[B57] Sánchez-López A. L. Ortiz G. G. Pacheco-Moises F. P. Mireles-Ramírez M. A. Bitzer-Quintero O. K. Delgado-Lara D. L. (2018). Efficacy of melatonin on serum pro-inflammatory cytokines and oxidative stress markers in relapsing remitting multiple sclerosis. *Arch. Med. Res.* 49 391–398. 10.1016/j.arcmed.2018.12.004 30595364

[B58] Sandyk R. (1993). Multiple sclerosis: the role of puberty and the pineal gland in its pathogenesis. *Int. J. Neurosci.* 68 209–225. 10.3109/00207459308994277 8063527

[B59] Sedel F. Bernard D. Mock D. M. Tourbah A. (2016). Targeting demyelination and virtual hypoxia with high-dose biotin as a treatment for progressive multiple sclerosis. *Neuropharmacology* 110 644–653. 10.1016/j.neuropharm.2015.08.028 26327679

[B60] Shannon R. J. Van Der Heide S. Carter E. L. Jalloh I. Menon D. K. Hutchinson P. J. (2016). Extracellular N-acetylaspartate in human traumatic brain injury. *J. Neurotrauma* 33 319–329. 10.1089/neu.2015.3950 26159566 PMC4761801

[B61] Sharman E. H. Bondy S. C. Sharman K. G. Lahiri D. Cotman C. W. Perreau V. M. (2007). Effects of melatonin and age on gene expression in mouse CNS using microarray analysis. *Neurochem. Int.* 50 336–344. 10.1016/j.neuint.2006.09.001 17118492 PMC1868445

[B62] Signoretti S. Marmarou A. Tavazzi B. Lazzarino G. Beaumont A. Vagnozzi R. (2001). N-Acetylaspartate reduction as a measure of injury severity and mitochondrial dysfunction following diffuse traumatic brain injury. *J. Neurotrauma* 18 977–991. 10.1089/08977150152693683 11686498

[B63] Srinivasan V. Spence D. W. Pandi-Perumal S. R. Brown G. M. Cardinali D. P. (2011). Melatonin in mitochondrial dysfunction and related disorders. *Int. J. Alzheimers Dis.* 2011:326320. 10.4061/2011/326320 21629741 PMC3100547

[B64] Steinman L. (1996). Multiple sclerosis: a coordinated immunological attack against myelin in the central nervous system. *Cell* 85 299–302. 10.1016/S0092-8674(00)81107-1 8616884

[B65] Tan D. X. Manchester L. C. Terron M. P. Flores L. J. Tamura H. Reiter R. J. (2007). Melatonin as a naturally occurring co-substrate of quinone reductase-2, the putative Mt3 melatonin membrane receptor: hypothesis and significance. *J. Pineal Res.* 43 317–320. 10.1111/j.1600-079X.2007.00513.x 17910598

[B66] Tress O. Maglione M. May D. Pivneva T. Richter N. Seyfarth J. (2012). Panglial gap junctional communication is essential for maintenance of myelin in the Cns. *J. Neurosci.* 32 7499–7518. 10.1523/JNEUROSCI.0392-12.2012 22649229 PMC6703577

[B67] Wanders R. J. Waterham H. R. Ferdinandusse S. (2015). Metabolic interplay between peroxisomes and other subcellular organelles including mitochondria and the endoplasmic reticulum. *Front. Cell Dev. Biol.* 3:83. 10.3389/fcell.2015.00083 26858947 PMC4729952

[B68] Welin A. K. Svedin P. Lapatto R. Sultan B. Hagberg H. Gressens P. (2007). Melatonin reduces inflammation and cell death in white matter in the mid-gestation fetal sheep following umbilical cord occlusion. *Pediatr. Res.* 61 153–158. 10.1203/01.pdr.0000252546.20451.1a 17237714

[B69] Yool D. Montague P. Mclaughlin M. Mcculloch M. C. Edgar J. M. Nave K. A. (2002). Phenotypic analysis of mice deficient in the major myelin protein Mobp, and evidence for a novel Mobp isoform. *Glia* 39 256–267. 10.1002/glia.10103 12203392

